# Examining predictors of non-suicidal self-injury among college students: A prospective cohort study

**DOI:** 10.1192/j.eurpsy.2025.599

**Published:** 2025-08-26

**Authors:** J. N. Kjaer, B. Turner

**Affiliations:** 1 Psychosis Research Unit, Aarhus University Hospital, Psychiatry; 2 Department of Clinical Medicine, Aarhus University, Aarhus, Denmark; 3 Department of Psychology, University of Victoria, Victoria, Canada

## Abstract

**Introduction:**

Non-suicidal self-injury (NSSI) refers to the deliberate act physical harm without intent of suicide. Common forms of NSSI include cutting, burning, and hitting oneself. The prevalence of NSSI among college students have been estimated to be between 15% to 25%.and higher than in the general population. Investigating patterns associated neurobiological and personality traits may provide a more comprehensive understanding of NSSI.

**Objectives:**

We aim to identify latent trajectory classes for NSSI behavior among college students. We expect that baseline personality and the behavioral inhibition/activation scales (BIS/BAS) will predict NSSI trajectory.

**Methods:**

A total of 704 first-year university students at University of Victoria, Canada, were recruited in the beginning of the first semester over two consecutive academic years. Participants attended a baseline testing session completing self-report measures including the Ten Item Personality Inventory, BIS/BAS and NSSI instruments. There were monthly follow-up sessions from October to April. Longitudinal data will be analysed with latent growth curve modeling and group-based trajectory modeling, and baseline predictors will be analysed with multivariate logistic regression.

**Results:**

Latent class growth analysis found three distinct classes of NSSI during the follow-up period. A small percentage (2.4%) of the participants had a high degree of self-injury throughout the follow-up period. A second class of 13.4% of the participants had a moderate degree of self-injury at baseline, which fell throughout the follow-up period. Lastly, a third class of the majority of the participants (84.3%) had minor or none self-injury both at baseline and in the follow-up period. Concerning baseline predictors, higher openness and BAS drive were associated with lower NSSI at baseline.

**Conclusions:**

In line with previous studies, we identified three distinct trajectories of NSSI behavior among college students. Notably, low openness and low BAS drive were associated with a degree of NSSI at baseline. These findings suggest that openness and drive may play a protective role in NSSI, providing valuable insights for future prevention and intervention efforts. The project is part of the Collaborative Research Program at the International Society for the Study of Self-Injury.

**Disclosure of Interest:**

None Declared

